# A Novel Training Program to Improve Human Spatial Orientation: Preliminary Findings

**DOI:** 10.3389/fnhum.2020.00005

**Published:** 2020-01-24

**Authors:** Michael McLaren-Gradinaru, Ford Burles, Inderpreet Dhillon, Adam Leonidas David, Alberto Umiltà, Jaimy Hannah, Kira Dolhan, Giuseppe Iaria

**Affiliations:** NeuroLab, Department of Psychology, Hotchkiss Brain Institute, and Alberta Children’s Hospital Research Institute, University of Calgary, Calgary, AB, Canada

**Keywords:** getting lost, hippocampus, memory, plasticity, rehabilitation, virtual reality

## Abstract

The ability to form a mental representation of the surroundings is a critical skill for spatial navigation and orientation in humans. Such a mental representation is known as a “cognitive map” and is formed as individuals familiarize themselves with the surrounding, providing detailed information about salient environmental landmarks and their spatial relationships. Despite evidence of the malleability and potential for training spatial orientation skills in humans, it remains unknown if the specific ability to form cognitive maps can be improved by an appositely developed training program. Here, we present a newly developed computerized 12-days training program in a virtual environment designed specifically to stimulate the acquisition of this important skill. We asked 15 healthy volunteers to complete the training program and perform a comprehensive spatial behavioral assessment before and after the training. We asked participants to become familiar with the environment by navigating a small area before slowly building them up to navigate within the larger and more complex environment; we asked them to travel back and forth between environmental landmarks until they had built an understanding of where those landmarks resided with respect to one another. This process repeated until participants had visited every landmark in the virtual town and had learned where each landmark resided with respect to the others. The results of this study confirmed the feasibility of the training program and suggested an improvement in the ability of participants to form mental representations of the spatial surrounding. This study provides preliminary findings on the feasibility of a 12-days program in training spatial orientation skills. We discuss the utility and potential impact of this training program in the lives of the many individuals affected by topographical disorientation as a result of an acquired or developmental condition.

## Introduction

Human topographical orientation is a complex behavior that serves a critical role in daily functioning: it provides the necessary skills to find the way around in both familiar and novel surroundings and allows individuals to move within the environment with a spatial purpose in mind (Marchette et al., 2011). Such a foundational skill could rely on different orientation strategies involving the use of environmental landmarks, as well as the memorization of given routes independently of landmarks encountered on the way (Siegel and White, 1975; Aguirre and D’Esposito, 1999). Among the many strategies adopted for orientation is the ability to form and make use of cognitive maps (Tolman, 1948). A cognitive map can be formed when an individual has gained configurable knowledge of an environment (understanding the location of landmarks in space with respect to each other) allowing the formation of a mental representation of the surrounding (Farran et al., 2015). This mental representation is formed as individuals become familiar with the surrounding, providing detailed information about salient environmental landmarks and their spatial relationships (Epstein et al., 2017). Cognitive maps are very critical to successful orientation since, once formed, they allow individuals to reach any target location from anywhere within the environment, and even permit generating alternative, unexplored routes if required by environmental circumstances (Arnold et al., 2013).

Cognitive maps are critical for orientation by enabling the planning of routes outside of the visible surroundings (i.e., vista space), which allows individuals to orient from all possible locations within a large-scale environment (i.e., environmental space; Montello, 1993; Wolbers and Wiener, 2014; Epstein et al., 2017). The formation and use of cognitive maps, however, is known to be affected by factors such as age and sex (Liu et al., 2011), as well as unknown factors that create a large amount of variability in the general population (Weisberg and Newcombe, 2018). For instance, in a very recent study, Yamamoto et al. (2019) compared the ability of young and older adults to learn an environment from both an aerial (top-down) perspective and a first-person perspective. They asked participants to watch videos showing a large-scale environment from a first-person perspective moving through the environment, an aerial view changing orientations, and an aerial view in a fixed orientation. The results confirmed that older adults learned an environment better from the fixed aerial perspective as compared to the first-person perspective, and from a fixed aerial perspective as compared to a rotating aerial perspective. This contrasts with young adults who revealed no difference in their ability to learn the environment between the three different perspectives. These results suggest that spatial strategies can differ throughout adulthood, where older adults may start to lose the ability to form new cognitive maps in a first-person perspective (Iaria et al., 2009b), likely due to structural changes in the hippocampus (Iaria et al., 2008), explaining the natural cognitive decline of topographical orientation skills occurring with aging (Wiener et al., 2013).

The critical role of cognitive maps for effective orientation and navigation in daily life is also confirmed by the lack of orientation skills as experienced by individuals affected by Developmental Topographical Disorientation (DTD; Iaria et al., 2009a; Bianchini et al., 2014; Barclay et al., 2016; Conson et al., 2018). DTD refers to a lifelong condition in which individuals get lost in extremely familiar surroundings despite well preserved general cognitive skills, no brain injuries, and no cognitive complaints (Iaria and Barton, 2010). As documented in many cases, the most common cause of topographical disorientation in individuals affected by DTD is the complete inability to form cognitive maps, confirming the important role of this skill for daily effective navigation (Iaria and Burles, 2016).

Despite the fairly well-known behavioral mechanisms underlying the ability to orient by means of cognitive maps, to date, very little is known about the potential effects of training programs that may help to improve such important skills. Although there is evidence that spatial skills are moderately malleable and could be improved by a specifically designed training program (Uttal et al., 2013), these studies have focused solely on improving performance on lower-level spatial functions such as mental rotation, visual selective attention, spatial problem solving, and landmark recognition (Subrahmanyam and Greenfield, 1994; Feng et al., 2007; Cherney, 2008; Lövdén et al., 2012; Nemmi et al., 2017; Xiao et al., 2018; Veurink and Sorby, 2019; Yamamoto et al., 2019). Consequently, this leaves a gap in the literature regarding whether or not it is possible to train the relatively higher-level ability to form cognitive maps. For example, Lövdén et al. (2012) analyzed the effects of a long-term virtual spatial training program on a variety of spatial outcome measures including mental rotation, route memory, and other lower-level spatial skills, but did not administer any tests that were designed to measure the ability to use cognitive maps. Additionally, because their training program utilized a constantly changing environment, it would have been difficult for a participant to develop an accurate, consolidated, cognitive map of the training environment at any given time. The latter highlights a second limitation found in previous literature, where training programs have been designed to train spatial skills other than cognitive map formation. Similarly, Montana et al. (2019) conducted a review on the use of virtual environments for rehabilitating spatial memory in stroke victims. Of the 16 studies analyzed in the review, none used a training program designed appositely to train cognitive map use, focusing instead on skills such as basic day-to-day living (e.g., go to the grocery store, shop for a list of groceries, pay with the right amount of money, and take the groceries home), route-based navigation, or exploration of small environments such as a house or grocery store. Consistent with previous literature, none of the 16 studies used any cognitive map outcome measures.

Here, we present a newly developed training program similar to those described in previous studied (e.g., Lövdén et al., 2012; Uttal et al., 2013; Montana et al., 2019), with the exception that this program was conceived specifically to improve the ability of the individuals to form cognitive maps in a video game-like virtual environment. To evaluate the feasibility of the training program, we asked participants to become familiar with the environment by navigating a small area before slowly building them up to navigate within the larger and more complex environment; we asked them to travel back and forth between environmental landmarks until they had built an understanding of where those landmarks resided with respect to one another. This process repeated until participants had visited every landmark in the virtual town and had learned where each traveled resided with respect to the others. A key component of this training program, described further in the methods section, is the unique task design aimed at mimicking the natural way cognitive map formation is acquired throughout development (Siegel and White, 1975). We expected that participating in the 12-days training program would result in a significant improvement of performance on a test measuring the specific ability to form cognitive maps (i.e., the Spatial Configuration Task, SCT; Burles et al., 2017). Additionally, we hypothesized that this difference would be greater than what would be found as a result of a test-retest effect in a non-training group.

## Materials and Methods

### Participants

We recruited 15 healthy volunteers (10 females; mean age = 34 years, ranging from 19 to 70 years), and referred to them as the “training group.” These individuals were recruited through advertisements on the University of Calgary website. After training participants in the experimental group, we recruited 23 participants (21 females, mean age = 20 years, ranging from 18 to 24 years) to be part of a *post hoc*, untrained group to control for any test-retest effects that may explain significant changes in spatial skills detected in the training group. These individuals were recruited through the resident psychology recruitment system at the University of Calgary where students are incentivized to partake in research studies for course credit. This group was only intended to address the testing effect on statistically significant changes detected in the training group. Thus, participants in this group only performed the SCT and did not complete any of the other tasks (i.e., the mental rotation and the Four Mountains Task; see descriptions below). Additionally, it should be noted that this group differs greatly in age range compared to the experimental group. Previous literature has shown that spatial skills are strongest in early adulthood, i.e., from ages 18–30 (Liu et al., 2011). With this in mind, we aimed to recruit individuals within this age range into the testing effect group in order to ensure that they had the highest chance of revealing a testing effect.

Participants in both groups were asked to complete a questionnaire reporting general demographics, neurological conditions or brain damage, anxiety (Marteau and Bekker, 1992) and depression (Kroenke et al., 2001) symptoms. We found that the training group did not vary significantly in the amount of years playing videogames compared to the TE group (*U* = 164, *p* = 0.788). A list of descriptive statistics for both groups can be seen in Table 1.

**Table 1:** Descriptive statistics for both the training and testing effect group.

	Training group	Testing effect group	Group difference	
	*M* (*SD*)	*M* (*SD*)	*U*	*p*
*N*	15, 5 males	23, 2 males		
Age in years	34.33 (19.17)	20.00 (1.73)		
Video game experience in years	4.07 (5.47)	2.44 (3.62)	164	0.788
Spatial Configuration Task				
Pre-training accuracy	0.48 (0.20)	0.55 (0.19)		
Post-training accuracy	0.62 (0.24)	0.62 (0.21)		
Four mountains				
Pre-training accuracy	0.62 (0.12)			
Post-training accuracy	0.69 (0.11)			
Mental rotation				
Pre-training accuracy	0.80 (0.10)			
Post-training accuracy	0.86 (0.09)			

Further, since there is no previous literature aimed at assessing the trainability of cognitive map formation, from a purely exploratory perspective we evaluated whether or not the training program would affect more specific spatial cognitive functions such as mental rotation and perspective-taking; to address this, participants in the experimental group were asked to complete two supplementary spatial tasks (i.e., the mental rotation task and the Four-Mountains Task; see description below) in the pre-training and post-training assessments.

#### Pre and Post-training Behavioral Assessment

Before and after the training program, participants in the training group were administered a battery of tests. The battery included the following series of tests aimed at assessing a variety of spatial skills that are important for orientation and navigation, including the ability to form cognitive maps.

*The Mental Rotation Task* is a well-known measure of spatial awareness and the ability to mentally represent and manipulate 3D images in one’s mind (Shepard and Metzler, 1971). The task consists of two abstract 3D objects placed side-by-side for the participant to compare and see if they are either the same object or two mirror images of each other (Vandenberg and Kuse, 1978). The objects are presented in varying spatial orientations, so the participant is required to mentally rotate the objects and determine if they are either “the same” or “different.” A total of 80 comparisons are made over the course of the task. Participants’ performance is scored on accuracy, where a higher amount of correct responses is considered a good performance on the task.

**Figure 1:**
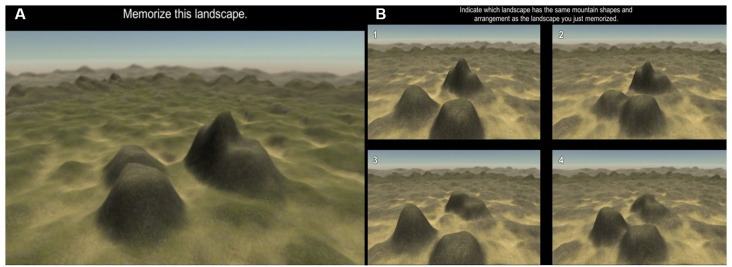
Example of a randomly generated scene from the Four Mountains Task **(A)**, and **(B)** recall phase of the task. In this example the correct answer is option #2.

*The Four Mountains Task* is designed to assess an individual’s perspective-taking abilities when presented with simple scenes (Hartley et al., 2007). In a trial, a scene containing a randomly generated set of four mountains is shown for 8 s, during which the participant is asked to memorize the scene to the best of their ability (Figure 1A). Following this, in the recall phase, participants are presented with four different pictures, each of four different sets of randomly generated mountains (Figure 1B). One of these four sets of mountains is the original set that the participant was been previously shown, however, this new picture was taken from an alternate angle or perspective. In addition, to remove the possibility of using the surroundings to determine the correct answer, both the lighting and texture of the terrain surrounding the mountains are changed. The task consists of 20 trials, each with a different set of randomly generated mountains. Participants’ performance is scored by measuring the number of correct responses such that a higher score represents better perspective-taking abilities.

**Figure 2:**
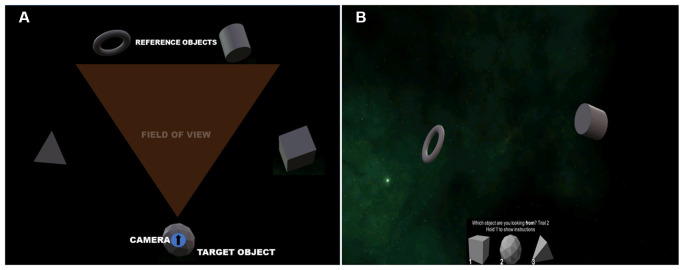
Overhead view of the Spatial Configuration Task (SCT; **A**), and **(B)** a sample trial in which the correct answer is object 2.

*The SCT* is designed to assess the ability to generate and use configural (configurable) knowledge (i.e., a mental representation of the relationship between objects in an environment, or a “cognitive map”) of geometric objects in an environment (Burles et al., 2017). Participants are presented with a space-like environment containing five abstract objects set in a pentagon (Figure 2A). Participants are not presented with the top-down view seen in Figure 2A, rather they are situated at one of the objects, facing inwards toward the center of the pentagon in a first-person view. This limits the participant’s view so they can only see two of the five objects in the pentagon (Figure 2B). They are then presented with the other three objects that are not in view as options to choose from at the bottom of the screen as seen in Figure 2B. At each of the 60 trials in this task, participants are required to choose the object they were looking from, or rather, situated on, from the objects presented at the bottom of the screen. Upon responding, the camera moves to a new object and the participant is required to repeat the steps described above. Participants are not given any direct feedback, however, while the camera is moving to a new object, it first turns and faces the object they were situated on, providing implicit feedback pertaining to their answer. Participants’ performance is scored on accuracy, where having a higher amount of correct responses indicates a better performance. Test-retest reliability conducted on the SCT has shown that it is internally consistent, but only within a single testing session (Burles et al., 2017).

### Training Program

#### Procedure

**Figure 3:**
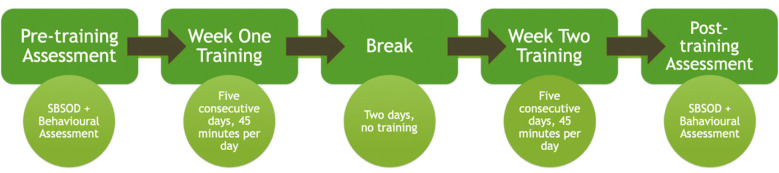
Timeframe of the training protocol across a 12-day period.

The training program begins by asking participants to complete the pre-training behavioral assessment, immediately followed by the first 45-min session in the training environment. Participants are then asked to complete eight additional 45-min sessions within the training program over a 10-days consecutive period, with a 2-days break after the first 4 days of training. Participants then complete a final 45-min training session followed by the post-training behavioral assessment. The total amount of training received is 10 45-min sessions across a 12-day period, with a 2-days break between the first five and last five sessions (see Figure 3). A 12-days training period was chosen based on the spatial training programs used in the previous literature. For instance, Meneghetti et al. (2016) used the Mental Rotation Task to train generalized spatial abilities where participants were asked to complete six, 45-min training sessions across a 2-week fixed period (three on week 1 and three on week 2) which was sufficient to produce lasting results on an object perspective task. Another study conducted by Wright et al. (2008) also used the mental rotation task to train generalized spatial abilities. In their study, participants were asked to complete 15–20 min of training each day over a 21-days training period, which was enough to improve reaction times on a non-trained spatial task. We chose our training program with the aim of being more intensive and over a shorter period of time than previous studies. Additionally, further support for this training period has been found in a very recent review by Montana et al. (2019) analyzing a variety of training programs using ecological virtual environments to train spatial skills in stroke patients. Results from this review showed beneficial results following 8–15 sessions lasting 40–45 min each, further suggesting that a semi-intensive (5 days per week) 12-days training period would be enough to produce lasting improvements.

#### Training Environment

**Figure 4:**
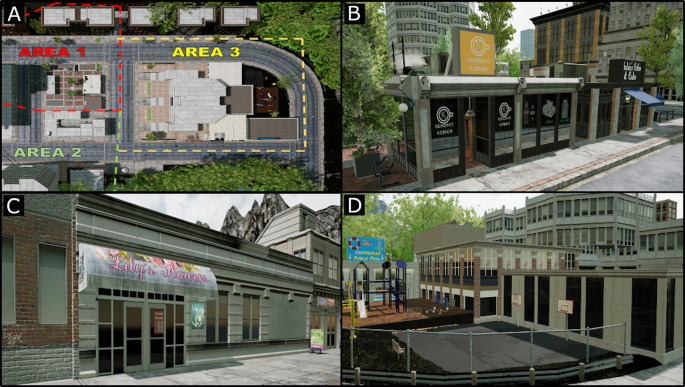
Different views of the virtual town “Centerville.” **(A)** Image showing how the training environment is split into three distinct areas. **(B)** Example buildings from Area 1, designed to be rustic with lots of colorful details making the buildings stand out. **(C)** Example buildings from Area 2, designed to be more industrial with gray colors making the buildings blend together. **(D)** Example buildings from Area 3, designed to be more modern and introduce more complex landmarks such as playgrounds, pools, and parking lots.

The training program takes place in a virtual environment that represents an urban neighborhood that we named Centerville. We designed Centerville using the Unreal Engine 4.15.3 under a Game developer’s license. We created the design for Centerville by researching common layouts of popular cities. Centerville was modeled after a curvilinear-loop design that integrates grid-like designs with curved lines. This allows for a combination of easier to navigate sections of the neighborhood in the grid section, with slightly more difficult sections of the environment outside of the grid. Centerville is split into three distinct areas that differ in the architecture and décor of the buildings as well as the types of landmarks present within the area (see Figure 4A). Area 1 has a rustic, brick-road look and contains stores, cafés, and restaurants (see Figure 4B). Area 2 has a washed-out, industrial look and contains office and government buildings (see Figure 4C). Area 3 is more diverse as it is designed to be the city center: it contains a city hall alongside multiple government office buildings (inside of which not available to participants for navigation), a community pool, and a recreational children’s park (see Figure 4D). Each of the three areas contains one “hub,” six major landmarks, and two to three minor landmarks. The hub of an area is the most outstanding landmark within the area and is used as an anchor to give participants a landmark that they can easily remember from each of the three areas. For example, the hub for Area 1 is the character’s apartment, the hub for Area 2 is the art gallery, and the hub for Area 3 is the main entrance to the large city hall building. The six major landmarks are stores, office buildings, and other amenities within a given area. The minor landmarks are landmarks that are not buildings, such as statues, billboards, and playground structures.

**Figure 5:**
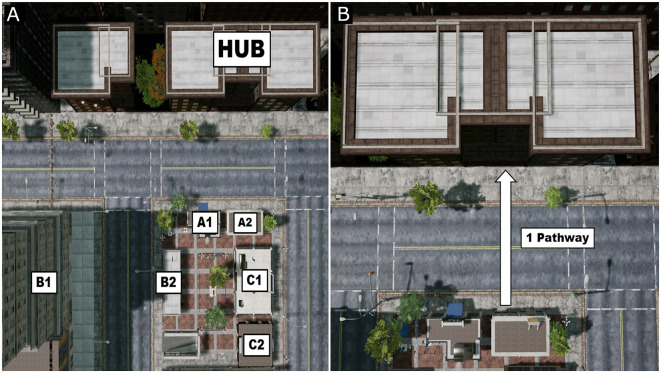
Division of areas in Centerville and example of a “pathway.” **(A)** Example of how each area is split into different components. Each area has a hub, two proximal, major landmarks directly near the hub (A1: Chelsie’s Coffee and A2: Kendra’s Korner Store), two proximal, major landmarks further away from the hub (B1: City Bank and B2: Atomic Clothes), and two proximal, major landmarks that are the furthest away from the hub (C1: Michael’s Bar and Grill and C2: The Liquor Store). **(B)** An example of a single pathway. A pathway can be as long or as short as necessary and is defined as the optimal path between the starting location and the target landmark.

Each of the three areas is divided into four additional sections: a section that includes the hub, and the three other sections each including two proximal, major landmarks (i.e., section A, B, and C; see Figure 5A). For example, referring to Area 1, the two proximal, major landmarks in Section A are the closest landmarks to the hub (e.g., Chelsie’s Coffee and Kendra’s Korner Store), making them the easiest to find when situated at the hub. The two proximal, major landmarks in Section B are slightly farther away from the hub (Atomic Clothes and City Bank), and generally take more effort to find and navigate to from the hub. Finally, the two proximal, major landmarks in Section C are the furthest away from the hub (e.g., Michael’s Bar and Grill and the Liquor Store) and take the most amount of effort to find. Each area also contains two to three minor landmarks: area 1 includes a statue of a man sitting on a bench and the Getting Lost billboard, Area 2 includes a definitive yellow bench and a children’s thrift store hidden in an alley, and Area 3 includes the Centerville Sphere statue, a large graffiti art in a tunnel, and a swing-set in the park.

Movement within the environment is achieved using a standard Xbox 360 controller. Participants control a first-person character and only have control of the forward/backward movement and the yaw (left and right turning). The movement is restricted as such to make movement easier for those with less video game experience. This movement is accomplished using the left joystick on the controller. Turning speed is kept at the default setting in the game engine and is smoothed using interpolation to avoid rapid starting and stopping of turning.

In order to practice the motor controls of the task, participants are asked to perform a practice session in a simple maze with one right and one left turn. Participants are instructed to move to the end of the maze without touching any walls and within a designated time period of 17 s. If a wall is touched or the participant takes too long, they are reset back to the beginning of the maze. When participants complete the maze correctly, they are presented with the training instructions and moved onto the training task.

#### Training Task

Throughout the entire training program, during each training session, participants perform as many trials as possible each asking them to reach a target landmark from a given starting location. Here, we use the term “pathway” to refer to the optimal path between the starting location and the target landmark (see Figure 5B). For each pathway, participants are required to perform a series of trials where they attempt to get from the starting location to the target landmark by traveling the shortest distance, as quickly as possible. This pathway is measured by the game engine, providing the optimal distance and time that could be traveled between the starting location and the target landmark, all while accounting for any obstacles (buildings, trees, signs, etc.). Naturally, the distances and times of the pathways vary according to the starting location and the complexity of the pathway to get to the target location. Based on a pilot study that we conducted, we account for a correct performance if participants travel to each target landmark within a 20% overage of the calculated optimal time and distance. Participants are required to perform three correct trials within a given pathway in order to move to a new pathway (i.e., a new starting location and target landmark). These correct trials do not have to be performed consecutively.

Upon reaching the target landmark, a participant receives one of three messages on the screen. If they successfully complete the pathway according to the criteria but have not yet completed three successful trials for the pathway, they are shown a message telling them to go back to the starting landmark (e.g., “Great job, now go to your apartment”). If this trial is their third successfully completed trial for the pathway, they get a message telling them to go find a new landmark (e.g., “Excellent, now go find Fresh Veggies”). If the participant reaches the target landmark but did not satisfy the distance or time criteria, they receive a message telling them to go faster while following the shortest path (i.e., “Good, now try to be faster while following the shortest pathway”); this message is intended to be ambiguous about whether they failed to reach the time or distance criteria so that the participant has to think about how to optimize their route as much as possible. If a participant does not reach the target landmark after a 400% window of the optimal time, they are presented with an arrow at the bottom of the screen pointing in the direction of the landmark (without providing any information of the pathway to be followed in order to get there); this gives the participant a hint of where the landmark is and encourages them to continue exploring on their own.

Participants completed six pathways (e.g., hub to A1, hub to B1, B1 to A1, B1 to C1, C1 to hub, C1 to A1) of major landmarks for each of the three areas. After completing six pathways in each area, they are asked to combine pathways across areas (e.g., hub (Area 1) to B1 (Area 2), B1 (Area 2) to the hub (Area 3), etc). After completing all combinations from these landmarks, they are asked to complete six more pathways between major landmarks that they did not travel the first time around in each of the three areas (e.g., hub to A2, hub to B2, etc). After completing these new pathways in each area, they are asked to combine these new pathways across areas [e.g., hub (Area 1) to B2 (Area 2), B2 (Area 2) to the hub (Area 3), etc]. Upon completing these pathways, they will have traveled every possible pathway between major landmarks in the game (784 pathways) across two full playthroughs of the game. If they reach this point, they are presented with a random pathway chosen from all major landmark pathways they have already completed, as well as the new minor landmarks they have not explored yet. We termed this “infinite mode” as the participant will keep getting a random pathway when they successfully complete a pathway. This will continue as such for the remainder of the training. During this mode, only one successful completion of the pathway is required to be given a new pathway.

### Data Analyses

All behavioral data were analyzed while accounting for variability in age, which has been shown to have a significant impact on spatial orientation skills (Iaria et al., 2009b; Uttal et al., 2013; Wolbers et al., 2014; Yuan et al., 2014; Coutrot et al., 2018; Yamamoto et al., 2019). For each behavioral measure collected from each participant in the training group, we conducted a one-way, repeated measure ANCOVA with two levels comparing pre-training and post-training scores using age as a covariate. We then conducted a repeated measures analyses of covariance (RMANCOVAs) comparing overall changes on pre-training and post-training scores in the SCT (measuring the ability to orient by means of cognitive maps), between the training group and the testing effect group using age as a covariate.

## Results

We first analyzed the assumptions for repeated measures analysis of covariances. All pre- and post-training measures satisfied the Shapiro–Wilk’s test of normality and were within a skewness range of −1 to 1. The covariate (age) was moderately correlated with the dependent variable (difference in SCT scores from pre to post) in the experimental group (*r*_(15)_ = −0.536, *p* = 0.04) but not in the testing effect group (*r*_(23)_ = −0.003, *p* = 0.987) suggesting that age would account for a sufficient portion of the variance in the experimental group. Additionally, the covariate did not violate the assumption of homogeneity of regression slopes between the experimental group and the testing effect group with a change in SCT scores as the dependent variable; *F*_(1,34)_ = 0.065, *p* = 0.801. No significant outliers were present in the data and as a result, all participants were included in the analysis.

### One Way Repeated Measure Analysis of Covariances

Three one-way repeated measures analyses of covariance (RMANCOVA) were conducted to determine the effect of assessment (pre-training and post-training) on the scores for each of the behavioral tasks while controlling for age in the training group. The analyses revealed a significant effect of assessment on the SCT accuracy after controlling for age (*F*_(1,13)_ = 5.234, *p* = 0.040, $\eta_{\text{p}}^{2}$ = 0.287). No significant effect of assessment was found on the Four Mountains Task (*F*_(1,13)_ = 0.204, *p* = 0.659, $\eta_{\text{p}}^{2}$ = 0.015), and the Mental Rotation Task (*F*_(1,13)_ = 0.141, *p* = 0.713, $\eta_{\text{p}}^{2}$ = 0.011). An additional one-way RMANCOVA was conducted to determine the effect of assessment (pre-training and post-training) on the calculated scores for the Santa Barbara Sense of Direction Scale while controlling for age in the training group. The analysis revealed that there was no significant effect of assessment on the scale after controlling for age (*F*_(1,13)_ = 1.798, *p* = 0.203, $\eta_{\text{p}}^{2}$ = 0.121).

**Figure 6:**
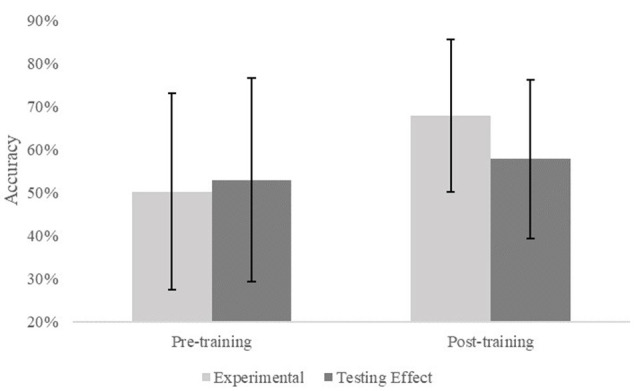
Estimated marginal means of the interaction effect from a repeated-measures analysis of covariance (RMANCOVA) conducted on the SCT comparing pre-training accuracy scores in the training group [*M* = 0.502, 95% CI (0.616, 0.388)] and testing effect group [*M* = 0.529, 95% CI (0.618, 0.440)] to post-training accuracy scores in the training group [*M* = 0.679, 95% CI (0.798, 0.561)] and testing effect group [*M* = 0.578, 95% CI (0.617, 0.486)] after accounting for age as a covariate. Error bars show 95% confidence intervals.

A two-way repeated-measures analysis of covariance (RMANCOVA) was conducted to test the hypothesis that the training group would perform better than the untrained testing effect group at the post-training assessment on the SCT while controlling for age. The analysis revealed a significant interaction effect between assessment (pre and post-training) and group (training and testing effect; see Figure 6) confirming our hypothesis (*F*_(1,35)_ = 6.380, *p* = 0.016, $\eta_{\text{p}}^{2}$ = 0.154). With a significant interaction found, follow up comparisons were conducted (Bonferroni corrected) to determine the source of the interaction: the only significant difference that was found was between the pre-training (*M* = 0.502, *SD* = 0.345) and post-training (*M* = 0.679, *SD* = 0.357) scores in the experimental group; *t*_(36)_ = 4.784, *p* < 0.001. All other comparisons were non-significant at an alpha level of 0.05.

## Discussion

This study reports the very preliminary findings of a newly developed training program specifically designed to improve the ability of the individuals to acquire configural knowledge of the spatial surrounding. The preliminary data available in this study confirm the feasibility of such a training program in the healthy population and seem to suggest an increase in the ability to form cognitive maps. This effect is in comparison to the testing effect group, who did not significantly improve in their scores on the SCT following a 12-days of no training period. It should be noted that the testing effect group was not a true control group and was only included in the study to control for the test-retest effect of the SCT across a 12-day period. The same training program did not result in any other significant improvements in task performance across other tests assessing cognitive functions such as mental rotation and perspective-taking. This effect could be explained by the nature of the SCT as it was appositely designed to measure the ability to create a mental representation of elements in space and their spatial relationships (Burles et al., 2017), a crucial, generalized skill in the formation and utilization of cognitive maps for orientation. In the SCT, participants are asked to identify the object (i.e., the location) that the camera is looking from. All objects are geometric abstract objects that are difficult to verbalize, making it much more difficult to remember them without directly visualizing them (e.g., “the round donut looking thing”) and consequently easier to simply visualize the objects. This means that to perform effectively and score high on the SCT, an individual must form a mental representation, or “cognitive map,” of the relationships between the five objects located in the surrounding. Participants’ improvement on the post-training SCT score will have suggested the development of a stronger ability to create and utilize cognitive maps for orientation.

Previous studies have examined the trainability of spatial skills in humans, providing good evidence that some spatial skills can be indeed improved by a training program (Uttal et al., 2013). While this is certainly important evidence, none of the studies reported in the literature have focused on specially training the ability to form cognitive maps. In the meta-analysis by Uttal et al. (2013), the authors examined 217 training studies focused on spatial skills, analyzing in detail the type of training (video game, course-based, and spatial task training) and the type of measure used for evaluating effective trainability of spatial skills. Although video games were a major theme in the meta-analysis, none of the measures reported were indicators of a person’s ability to form cognitive maps, which is consistent with more recent studies. For instance, a study by Xiao et al. (2018) examined the potential for a video game platform to train spatial visualization skills without using any measures to determine improvements in cognitive map formation abilities. Similarly, Veurink and Sorby (2019) recently analyzed the results of a longitudinal study aimed at improving spatial skills in engineering students who initially scored low on the Purdue Spatial Visualization Test, a measure of one’s mental rotation ability (Bodner and Guay, 1997). In this study, the training program focused on several spatial skills, such as the rotation of objects and cutting planes, but none of their measures focused on cognitive map formation (Veurink and Sorby, 2019). While no studies to date have examined the potential to train the ability to form cognitive maps, it remains difficult to argue that training specific spatial skills (such as mental rotation or landmark recognition) would result in better spatial orientation and navigation. Our present study with a newly developed tool that allows testing the hypothesis that high-cognitive level functions in the spatial domain can be trained, and potentially result in improved spatial orientation skills in people’s daily lives.

The evidence that cognitive maps are a trainable skill could have important implications for improving the lives of many individuals. First, individuals affected by DTD have a selective inability to orient in very familiar surroundings due to their inability to form cognitive maps (Iaria and Burles, 2016). Cognitive map training, as described in our study, could directly target this missing skill in people with DTD, leading to an improvement in their navigation abilities and subsequently raising their quality of life. Second, aging has been shown to reduce the effectiveness of navigation in the elderly as it seems to cause a switch from survey-based navigation to less effective route-based navigation strategies (Yamamoto et al., 2019). The ability to train cognitive map skills could keep survey-based navigation as the main strategy in older adults, potentially preserving effective navigation skills in novel surroundings throughout old age. In addition, as shown by Lövdén et al. (2012), navigation training can protect against age-related changes to the hippocampus in both young and older adults. This implies that hippocampal-dependent spatial training, such as training cognitive map formation, may produce neuroprotective benefits leading to healthier brain aging. Finally, recent studies have suggested that spatial navigation quality can be used as an early marker for Alzheimer’s disease, a disease that typically starts with degeneration of the hippocampus (Coughlan et al., 2018; Laczó et al., 2018; Parizkova et al., 2018). Alzheimer’s patients commonly show orientation issues (Coughlan et al., 2018), suggesting that the early stages of Alzheimer’s can be diagnosed with a decline in orientation skills. A training program effective at improving spatial orientation skills such as the one described in our study could have a significant impact on delaying the degenerative process related to the decline of spatial orientation skills in Alzheimer’s disease. However, our study does not provide evidence on the efficacy of the training program in any of these populations and thus these speculations should be taken with caution.

Interestingly, we found that scores on the mental rotation and Four Mountain Tasks were not significantly affected by the training program. Although it may seem surprising at first, this is expected given that the mental rotation task is designed to assess the ability to mentally rotate small 3D objects in a scale of space known as *figural space* (Hegarty et al., 2006). Figural space is a scale of space that is external to the individual, can be fully observed from a single viewpoint, and is occupied only by a single object such as a car or a coffee cup (Hegarty et al., 2006). Figural space is smaller than *vista space* which can be fully observed from a single viewpoint but is occupied by multiple environmental elements (objects, landmarks, landscapes, etc.) to create a scene. Figural space also differs from the so-called *environmental space*, which refers to a large environment that can only be fully observed from multiple, different, angles and viewpoints (Hegarty et al., 2006). An individual would need to navigate environmental space to become familiar with it, in comparison to vista and figural space which can be fully observed while standing still. While visualizing and manipulating figural space is important for cognitive map formation and utilization, it is only a small component of the overarching skill. Therefore, one would expect the effects of a training program to be visible on the general ability of cognitive map formation and usage, rather than the individual components of it. The same applies to the lack of effects found in the Four Mountains Task, as this task assesses perspective-taking, another individual component of the ability to form cognitive maps (Hartley et al., 2007).

The main aim of our study was to present a newly developed cognitive training potentially effective in improving the ability of the individuals to form cognitive maps. The preliminary findings reported in this study have some significant limitations that are important to be aware of before making any conclusion on the efficacy of such a program. First, we have collected data on small sample size, and it would be very valuable to test a larger population of individuals to improve the power of the study. Ideally, a future study should be also balanced in sex across the groups, as the current study sample is low on male participants. Sex is known to have a significant effect on spatial abilities, with a specific effect on the ability to form cognitive maps (Moffat et al., 1998; Astur et al., 2004; Iaria et al., 2009b; Liu et al., 2011; Fernandez-Baizan et al., 2019). Thus, in the context of cognitive map formation abilities, keeping the groups balanced can help to remove unexplained variance due to sex from the sample, and investigate the effectiveness of the training program across sex. Similarly, gathering a larger sample size while keeping a broad range of ages will lead to a better understanding of the effect that age may have on the benefits gained from the training program. In addition, a future study should evaluate the long-term effects of the training program on the daily life orientation skills of the individuals; this will require a longitudinal study given that the skills acquired through the training program may require some time and practice to be consolidated in the real-life navigation. Moreover, it is worth noticing that participants in our control group were not asked to complete a non-spatial training program, and the group was included to control only for the passage of time and for repetition effects of the SCT. To overcome this limitation, a future study should include a control group in which participants are asked to perform a daily non-spatial training program with an experimental protocol identical to one followed by the training group. Finally, the lack of follow up measurements does not provide us with information regarding the long-term effects of the training program; without such evidence it would be difficult to prove that the training could have a significant impact on people’s daily life. For all reasons described above, the findings reported in our study should be treated as providing preliminary evidence for an effective program aiming to train the ability to form cognitive maps for orientation. Nevertheless, these preliminary findings, together with previous work available in the literature, seem to point towards a very important training opportunity for improving the ability to orient in large-scale surroundings. This has the potential to be beneficial to a variety of individuals suffering from topographical disorientation due to a developmental or acquired condition. However, the limitations in this study cause the results to be too speculative to apply training programs to these populations at this stage. Further testing on healthy subjects using a study design with a true control group will be required before more confident conclusions can be made on the efficacy of this training program.

## Data Availability Statement

The datasets generated for this study are available on request to the corresponding author.

## Ethics Statement

The studies involving human participants were reviewed and approved by Conjoint Faculty Research Ethics Board—University of Calgary. The patients/participants provided their written informed consent to participate in this study.

## Author Contributions

All Authors contributed significantly to the work presented in this manuscript and approved the final version of the manuscript. MM-G, FB, ID, AU, and GI conceived the training program and developed the experimental protocol. MM-G, KD, and AL performed data collection. MM-G, FB, JH, and GI performed data analyses. MM-G prepared the first draft of the manuscript.

## Conflict of Interest

The authors declare that the research was conducted in the absence of any commercial or financial relationships that could be construed as a potential conflict of interest.
